# Scirrhus of the Uterus Complicated with Pregnancy; Funis Presentation; Delivery

**Published:** 1853-02

**Authors:** 


### OBSTETRICS.

*Scirrhus of the Uterus complicated with Pregnancy; Funis presentation; Delivery.*—Mr. I. Brown read the following case: Mrs. W——, aged thirty-five, an out-patient of St. Mary's Hospital, was under my care from May to July with scirrhus of the os and cervix uteri. The catamenia had ceased for several weeks, but there was a good deal of offensive serous discharge. Her health became so seriously injured, that she was unable to continue her attendance as an out-patient, and I lost sight of her until November 18th, when I was requested by Mr. Hammond, one of the district accoucheurs of the Maternity, to visit her. I found that she had been seized with symptoms of labor the previous day, that the waters had escaped, and that the funis had descended six or eight inches through the os externum; but the thickened edges of the uterus seemed quite unyielding, and the labor made little or no progress. The patient became excessively exhausted, requiring ammonia, brandy, and strong beef-tea. I suggested that they should wait and see if the os dilated a little more, and if it did that delivery should be attempted by turning. Turning was accomplished the following day by Mr. Bullock, resident-surgeon to St. Mary's Hospital, and Mr. Hammond, after considerable difficulty. The fœtus was one of seven months, and had been dead some five or six days. The patient remained in a very exhausted state after her delivery, and when Mr. Brown last saw her, was evidently fast sinking.

In answer to questions, Mr. Brown could not say how long the disease had existed, but he supposed for some time. About half the os was involved in the ulceration.

Dr. Routh recollected the case of a woman in Vienna, in whom three fourths of the os were involved in the ulceration. The woman was four days in labor, and died four or five days after delivery. The delivery was effected, not through the os, but on one side of it. The woman died of peritonitis.

Dr. Barnes said that the practical question in this case was, as to the propriety of effecting premature delivery to avoid the passage of a large body through the diseased os. Would such a proceeding give the patient a chance of recovery? He thought that the cases of Dr. Rowland had proved the contrary, and that as death would result whenever delivery took place, life of course would be prolonged by allowing the woman to go the full time.

